# Psychobiotics at the Frontiers of Neurodegenerative and Neuropsychiatric Research

**DOI:** 10.3390/microorganisms13122718

**Published:** 2025-11-28

**Authors:** Guillermo Roberto Jiménez-Pareyón, José Melesio Cristóbal-Luna, Yuliana García-Martínez, Cynthia Garfias-Noguez, Morayma Ramírez-Damián, Edgar Torres-Maravilla, María Elena Sánchez-Pardo

**Affiliations:** 1 Instituto Politécnico Nacional, Escuela Nacional de Ciencias Biológicas—Campus Zacatenco, Unidad Profesional Adolfo López Mateos, Zacatenco, Av. Wilfrido Massieu 399, Colonia Nueva Industrial Vallejo, Alcaldía Gustavo A. Madero, Mexico City 07738, Mexico; guillermo.jimenezpareyon@gmail.com (G.R.J.-P.); josmcl@hotmail.com (J.M.C.-L.); ygarciamart@hotmail.com (Y.G.-M.); cgarfez@gmail.com (C.G.-N.); mramirezd1601@alumno.ipn.mx (M.R.-D.); 2 Facultad de Medicina y Nutrición Mexicali, Universidad Autónoma de Baja California, Mexicali 21000, Mexico

**Keywords:** probiotics, psychobiotics, neurodegenerative disease, neuropsychiatric disorders, gut microbiota-brain axis

## Abstract

Neurodegenerative and neuropsychiatric disorders remain a major public health concern due to their progressive nature, high prevalence, and considerable socioeconomic burden. Conventional treatments often fall short, facing limitations such as pharmacoresistance, adverse effects, and limited efficacy, underscoring the need for complementary approaches. Recent advances highlight the central role of the gut–brain axis (GBA) in neurological health, positioning psychobiotics and probiotic strains with potential mental health benefits, as candidates in adjunctive therapy. This review integrates current evidence on the GBA’s involvement in conditions such as Alzheimer’s disease, Parkinson’s disease, depression, and anxiety. We examine how psychobiotics may modulate neuroinflammation, oxidative stress, and neurotransmitter signaling, thereby contributing to cognitive and emotional regulation. Both preclinical and clinical studies are discussed, with emphasis on biomarker changes, quality-of-life outcomes, and neuropsychiatric comorbidities. We also explore recent innovations, including precision psychobiotics, microbiota–drug synergies, and their relevance to overlapping metabolic and neurodegenerative pathologies. Finally, we address the major translational challenges in the field, strain selection, methodological standardization, biomarker integration, and ethical design, highlighting key perspectives for advancing psychobiotics research toward clinical application.

## 1. Introduction

Neurological diseases, such as depression, anxiety, Alzheimer’s disease (AD), and Parkinson’s disease (PD), pose a growing challenge for global public health. These conditions not only diminish the quality of life for millions of people but also impose significant economic and social costs [1]. Their increasing prevalence is strongly associated with population aging, the principal risk factor for most neurodegenerative diseases and a contributor to the onset and progression of psychiatric conditions [2]. This scenario underscores the need for innovative and complementary therapeutic approaches that go beyond conventional pharmacological treatments [3].

Current treatments for neurodegenerative and neuropsychiatric disorders are often limited by symptomatic focus, modest efficacy, adverse effects, and lack of long-term disease-modifying capabilities. Moreover, the multifactorial nature of these disorders—encompassing genetic, environmental, immunological, and metabolic dimensions—necessitates the development of broader therapeutic perspectives. Many conventional pharmacological treatments, including antidepressants, have been shown to disrupt the gut microbiota, contributing to dysbiosis and increasing the risk of associated health issues, such as *Clostridioides difficile* infections [4]. Long-term use of antidepressants, especially in older adults, has been linked to adverse effects including falls, hyponatremia, stroke, and gastrointestinal disturbances. Furthermore, several medications modulate microbial composition in ways that may either enhance or hinder therapeutic outcomes [5]. These findings underscore the importance of integrative approaches to mental and neurological health, including psychological support, nutritional strategies, and potentially microbiota-based interventions such as psychobiotics.

Over the last decade, growing scientific evidence has highlighted the importance of the intestinal microbiota in human health and its influence on various physiological functions, including the regulation of the central nervous system [6]. This bidirectional communication system, known as the gut–brain axis (GBA), involves neural, endocrine, and immune pathways that enable the gut microbiota to modulate brain activity, mood and behavior [7]. Within this framework, the concept of psychobiotics has gained attention [8]. Originally introduced in 2013 [7], psychobiotics are defined as live organisms that, when ingested in adequate amounts, confer mental health benefits by modulating the GBA [8]. These benefits are thought to be mediated through mechanisms, including modulation of neurotransmitter synthesis (e.g., γ-aminobutyric acid (GABA), serotonin), immune and inflammatory signaling, oxidative stress reduction, and regulation of hypothalamic–pituitary–adrenal (HPA) axis responses.

Given the increasing interest in microbiota-based interventions, psychobiotics represent a promising adjunctive approach for the management of both neurogenerative and neuropsychiatric conditions. This review explores the potential of psychobiotics to influence the pathophysiology of these disorders by integrating evidence of both representative preclinical and clinical studies. Considering the multifactorial etiology of these disorders, including genetic, inflammatory, and environmental factors, psychobiotics offer a complementary strategy that may interact with several physiological pathways. The discussion integrates relevant concepts from microbiological, neurological, and behavioral sciences to better understand the role of psychobiotics in brain health and disease.

## 2. Pathophysiology of Neurodegenerative and Neuropsychiatric Disorders

To explore the potential therapeutic role of psychobiotics, it is first essential to understand the underlying mechanisms of neurodegenerative and neuropsychiatric disorders. These conditions encompass a broad range of diseases that affect the central and peripheral nervous systems, often leading to progressive deterioration in cognitive, emotional, and motor functions [9]. They are typically characterized by synaptic loss, neurotransmitter imbalances, neuroinflammation, and other pathological changes that manifest clinically as cognitive decline, emotional dysregulation, muscle weakness, or even altered states of consciousness [10,11,12].

The International Classification of Diseases (ICD) provides a structured taxonomy of neurological disorders based on their etiology, anatomy, location, and symptoms. According to this framework, neurological diseases are defined as a group of conditions located in or associated with the nervous system [13,14,15]. For the purposes of this review, we focus on neurodegenerative disorders, specifically Alzheimer’s disease and Parkinson’s disease, as well as psychiatric and mood disorders such as depression and anxiety.

### 2.1. Neurodegenerative Diseases and Their Classification

Neurodegenerative diseases (NDs) are a group of disorders characterized by the progressive deterioration of the central or peripheral nervous system. These diseases cause morphological changes in the brain, leading to significant cognitive or motor impairments, debilitating symptoms, and a reduced quality of life [16]. NDs involve complex cellular responses triggered by the accumulation of pathologically altered brain substances, ultimately resulting in irreversible loss of neuronal populations [17].

The pathophysiology of NDs is multifactorial and intricate, involving cellular, molecular, and genetic mechanisms. These include protein misfolding and aggregation, oxidative stress, mitochondrial dysfunction, cytoskeletal abnormalities, disrupted synaptic networks, neuronal death, aberrant cell proliferation, neuroinflammation, demyelination, altered axonal transport, dysregulated energy metabolism, and abnormal modifications of DNA or RNA [16,18,19,20,21,22].

**Figure 1:**
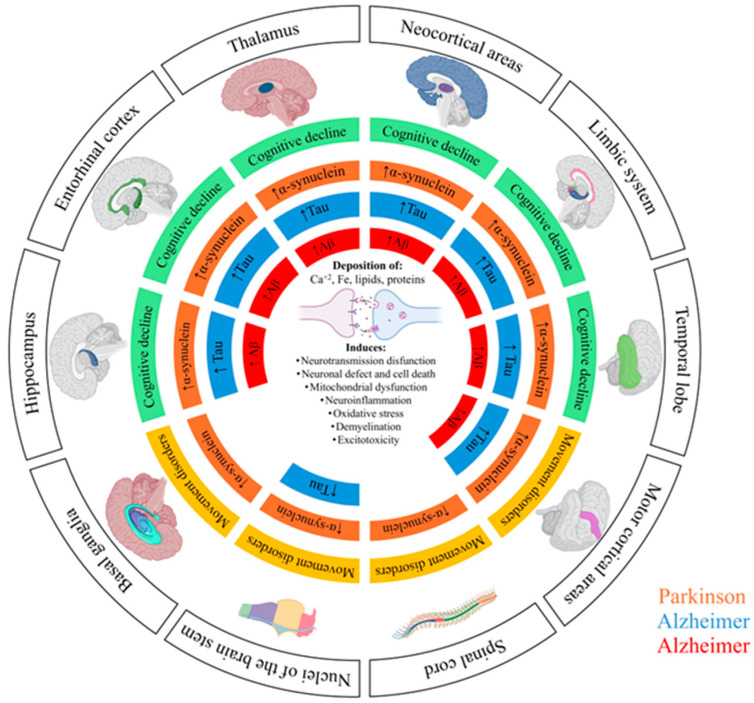
Schematic representation of the pathological progression of Alzheimer’s disease (AD) and Parkinson’s disease (PD) across different brain regions. The diagram illustrates the spatial distribution and accumulation of characteristic protein tau (Tau highlighted in blue) and β-amyloid (Aβ highlighted in red) in AD, and α-synuclein (α-synuclein highlighted in orange) in PD. These pathological protein aggregates are associated with multiple cellular processes, including neurotransmission dysfunction, neuronal loss and death, mitochondrial dysfunction, neuroinflammation, oxidative stress, demyelination, and excitotoxicity. The colored areas indicate the brain regions affected in each disease, highlighting the relationship between protein accumulation and the onset cognitive (green) or motor symptoms (yellow).

NDs can be classified according to several criteria, such as their etiology, the molecular mechanisms involved, and the anatomical regions affected [23]. Although multiple classification systems exist, these disorders often share overlapping cellular and molecular mechanisms [24], which complicates efforts to categorize them into a single scheme. From a mechanistic perspective, NDs commonly exhibit recurring pathological events such as neuroinflammation, oxidative stress, mitochondrial dysfunction, and the accumulation of misfolded proteins [16]. Recent classification systems increasingly emphasize the type of protein aggregates for diagnostic accuracy [25]. Clinically, NDs can also be classified clinically based on predominant symptoms, such as movement disorders in PD and Huntington’s disease, cognitive deficits in AD, or a combination of both [12,26,27]. This approach allows for a better understanding of their heterogeneity and facilitates the development of more specific therapeutic strategies. Among the spectrum of neurodegenerative conditions, AD and PD are the most prevalent and extensively studied (Figure 1).

### 2.2. Physiopathology Associated with Neurodegenerative Disorders

A defining feature of NDs is the progressive loss of brain cells over time, driven by diverse molecular and cellular mechanisms that impair neural function [28]. Understanding these pathophysiological factors is fundamental for analyzing the decline in patient health [29]. Prioritizing the most studied and relevant mechanisms, especially those pertinent to psychobiotics modulation, can aid in designing more effective interventions. Key mechanisms include:

- Protein aggregation: In many NDs, the abnormal accumulation of misfolded proteins disrupts cellular function and contributes to neuronal toxicity. These aggregates interfere with physiological processes and exacerbate neuronal dysfunction [30].
- Cellular dysfunction: NDs often involve the selective loss or aberrant proliferation of specific neural cell types. Both neurons and glial cells can experience alterations that compromise their function, including the loss of synaptic communication, dysregulation of intracellular transport mechanisms, and the inability to maintain ionic and energetic homeostasis, all of which accelerate degeneration [31].
- Biochemical imbalances: Dysregulated biochemical processes can induce cellular dysfunction and, eventually, neuronal death and the accumulation of neurotoxic metabolites that contribute to neural injury [32].
- Genetic factors: Mutations in key genes (e.g., APP, PSEN1 and PSEN2, PARK7 (DJ-1), PINK1, and PRKN) increase susceptibility to NDs by interfering with critical pathways related to protein processing, mitochondrial integrity and autophagy [33,34].

While neuroinflammation may not initiate these disorders, it significantly amplifies disease progression. Chronic activation of microglia and sustained inflammatory signaling can exacerbate neuronal damage and accelerate disease development. This inflammatory response arises from primary pathological events such as protein aggregation, cellular dysfunction, and biochemical alterations, making it an important target for therapeutic strategies.

### 2.3. Psychiatric and Mood Disorders

The WHO defines mental health as a state of well-being where certain aspects are included: emotional, social, psychological and clinical, allowing individuals to maintain functional lives even under stress [35].

From a psychiatric perspective, mental health can be understood as a balanced state that allows individuals to utilize their cognitive, emotional, and social skills to solve problems. This concept includes physical health, expression abilities, emotional intelligence, and the connection between mind and body [36].

Currently, millions of people worldwide experience mental health decline and are diagnosed with psychiatric disorders. These disorders are medical conditions in which cognition, emotional regulation, behavior, or psychological functioning are affected. Psychiatric disorders often involve neural circuit dysfunction, neurotransmission abnormalities, and even structural or functional brain alterations [37].

Anxiety and depression have emerged as major mental health conditions, with an alarmingly increasing prevalence across countries, cultures, and social strata. These disorders are not merely mood fluctuations but complex conditions that significantly impair quality of life [38].

### 2.4. Need for Complementary Approaches in Neuroprotection

Neuroinflammation and oxidative stress are central mechanisms in the pathophysiology of neurodegenerative and neuropsychiatric diseases, contributing to neuronal loss, altered neurotransmission, and cognitive or behavioral decline. These processes are sustained by excessive glial activation, cytokine release (e.g., TNF-α, IL-1β, IL-6), blood–brain barrier (BBB) dysfunction, and the infiltration of peripheral immune cells into brain tissue. Key biomarkers such as beta-amyloid (Aβ), tau protein, α-synuclein, and inflammatory cytokines are widely used to assess disease progression in Alzheimer’s disease (AD), Parkinson’s disease (PD), depression, and anxiety [39,40,41,42,43,44,45]. Despite advances in pharmacological therapies, high treatment costs, resistance, limited accessibility, and potential side effects underscore the need for innovative, integrative approaches to neuroprotection. Natural compounds with antioxidant and anti-inflammatory properties, as well as interventions targeting the GBA, are gaining attention as promising complementary strategies [46].

Among these, compounds with antioxidant and anti-inflammatory activity derived from natural sources display promising neuroprotective effects [47]. The gut–brain axis has also emerged as a key modulator of brain health, with increasing evidence linking gut dysbiosis to neuroinflammation and cognitive decline. Psychobiotics, a subclass of probiotics targeting the GBA represents a promising avenue. These microbes not only mitigate neuroinflammation but also influence neurotransmitter synthesis (e.g., 5-HT, GABA), regulate HPA axis activity, and support synaptic plasticity. Through the production of short-chain fatty acids (SCFAs) and other signaling metabolites, psychobiotics offer a mechanistic basis for modulating neuroimmune interactions and improving both cognitive and emotional outcomes [48,49]. The dual action of psychobiotics addressing both neurodegenerative and neuropsychiatric components makes them a valuable complement to existing therapies. Therefore, their inclusion in integrated treatment paradigms could enhance neuroprotection, improve quality of life and reduce the economic burden of these conditions.

## 3. The Gut–Brain Axis (GBA): Mechanisms and Communication Pathways

The GBA represents a bidirectional communication system between the gastrointestinal tract and the central nervous system, mediated by neural, hormonal, and immune functions [50]. Importantly, disruptions in this pathway, particularly gut dysbiosis, defined as an imbalance in gut microbiota composition, have been linked to various psychiatric and neurological conditions [51]. Emerging evidence suggests that alterations in gut microbiota can influence brain function through multiple mechanisms, including modulation of the HPA axis, which regulates stress responses [52]. Altered GABA signaling, the main inhibitory neurotransmitter pathway in the central nervous system, has been consistently associated with anxiety, depression, and cognitive dysfunction. Both microbial imbalance and host dysregulation can reduce GABA availability, contributing to these disorders. Supplementation with high GABA-producing bacteria, such as *Lactiplantibacillus* (*Lpb.*) *plantarum* L5, or exogenous GABA administration has been shown to restore GABA levels, reduce inflammatory cytokines (IL-1*β*, IL-6, TNF-*α*) and ameliorate behavioral and neuroinflammatory alterations in experimental models [53,54].

Alongside neurotransmitters, microbial metabolites such as SCFAs (e.g., butyrate, propionate, acetate) play key roles in gut–brain communication and neuroimmune regulation. Accordingly, interventions using probiotics (e.g., Lactobacilli strains) and prebiotic fibers have been shown to restore microbial balance, enhance SCFA production, and support cognitive and neuroendocrine functions [55]. In PD, a consistent and significant reduction in SCFA-producing bacteria has been observed across multiple cohorts, correlating with increased gut permeability and systemic inflammation, suggesting a potential link between microbial alterations and neurodegenerative progression. A large-scale metagenomic analysis by Wallen et al. [56], involving 490 PD patients and 234 controls reported a marked depletion of key SCFA producers, *Roseburia intestinalis*, for example, was reduced 7.5-fold, alongside enrichment of opportunistic taxa such as *Bifidobacterium dentium*, *Streptococcus mutans*, and *Actinomyces oris* [56]. Similarly, Aho et al. [57], observed significant microbial differences between PD patients and controls across two timepoints, including reduced *Prevotella* abundance in faster-progressing individuals. These microbial shifts formed distinct dysbiotic clusters, with antagonistic interactions between enriched and depleted species, suggesting competitive exclusion dynamics.

Dietary components, such as tryptophan and tyrosine (precursors of serotonin and dopamine), can also be metabolized by gut microbes, potentially influencing neurotransmission and behavior. Microbial catabolism of these amino acids yields a variety of neuroactive compounds, including indoles and phenolic derivatives, that may act locally or systemically. However, the precise molecular mechanisms of gut–brain communication remain under active investigation [58].

### Mechanisms of Gut–Brain Communication

**Figure 2:**
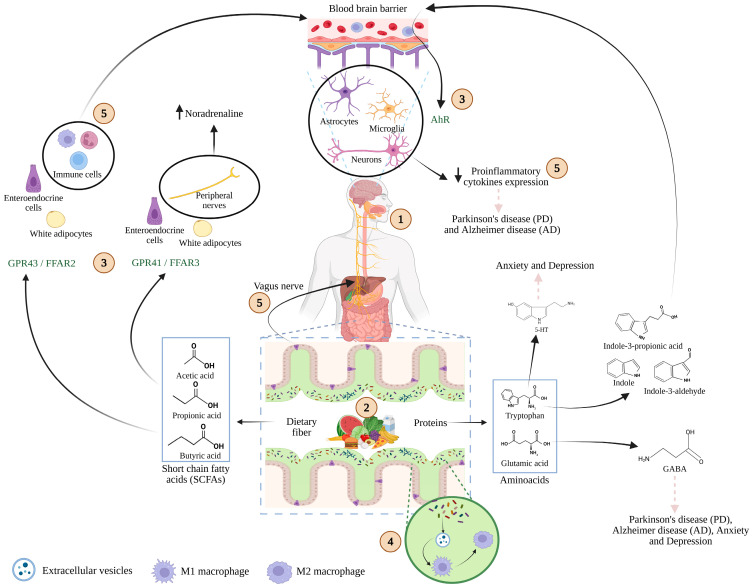
Schematic representation of the microbiota–gut–brain axis. (1) Food intake delivers key nutrients, including carbohydrates, lipids, proteins, vitamins, and minerals that are metabolized not only by the host but also by gut microbiota. (2) Microbial fermentation of these macronutrients generates bioactive metabolites such as short-chain fatty acids (SCFAs) and amino acid derivatives. (3) These compounds interact with specific host receptors, including G-protein-coupled receptors (GPR43/FFAR2, GPR41/FFAR3) and the aryl hydrocarbon receptor (AhR), which are expressed on epithelial, immune, and neural cells. (4) Extracellular vesicles derived from microbiota play a pivotal role in macrophages modulation. (5) Through these interactions, microbial metabolites trigger intracellular signaling cascades, modulate immune responses, and influence central and peripheral processes involved in health and disease. Adapted from [62,63,64]. Created with BioRender.com (2025) (accessed on 17 November 2025).

The GBA is composed mainly of three components (Figure 2): (i) the first is the gut microbiota, a complex ecosystem of bacteria, viruses, and fungi, with bacterial populations receiving the most studied. Advances in microbiome research have revealed how microbial genes influence host metabolism and immune signaling [59]. (ii) The second is the gastrointestinal tract, where enterocytes and the mucus layer serve as both a selective barrier and interactive interface, allowing beneficial microbes to thrive and participate in host immune and metabolic functions [60]. The third is (iii) the enteric nervous system (ENS), composed of neurons, interneurons, motor neurons, and glial cells arranged in myenteric and submucosal plexuses. Although it can operate autonomously, it also communicates with the central nervous system (CNS) [61].

Understanding how this communication occurs has been the focus of intensive research, particularly regarding microbial metabolites. Butyrate and propionate act as ligands for G-protein-coupled receptors (GPR41/FFAR3 and GPR43/FFAR2); the latter is expressed in immune cells capable of crossing the blood–brain barrier (BBB) and regulate microglia. Amino acids and their derivatives are also pivotal. For instance, the tryptophan metabolite serotonin (5-HT) is implicated in anxiety and depression, restoring 5-HT levels can ameliorate these conditions. Indole compounds derived from tryptophan can cross the BBB and bind to the aryl hydrocarbon receptor (AhR) on neurons and glial cells modulating inflammatory cytokines relevant to AD and PD. Similarly, GABA, a derivate of glutamate, acts as a key inhibitory neurotransmitter and has immunomodulatory properties with relevance to mood and neurodegenerative disorders [62,63,64]. In a recent 12-week double-bind, placebo-controlled trial, Decaya et al. [65], explored the effects of a multi-strain probiotic formulation (*Limosilactobacillus fermentum* LF16, *Lacticaseibacillus rhamnosus* LR06, *Lactiplantibacillus plantarum* LP01 and *Bifidobacterium longum* 04), on neurotransmitter levels. They reported a transient increase in plasma serotonin (5-HT) concentrations by week 6; followed by return to baseline by week 12. In contrast, plasma GABA levels remained stable throughout the trial. The authors suggest that this may reflect a microbiota-mediated feedback mechanism regulating host serotonin synthesis.

Beyond classical neurotransmitters, recent studies have emphasized the role of bacterial extracellular vesicles (EVs) as interkingdom signaling agents. Li et al. [66] demonstrated that EV concentration in Lactobacilli strains isolated from kefir modulates extracellular polysaccharides (EPS) production and biofilm formation. On the other hand, Fan et al. [67], showed that EVs derived from *Ligilactobacillus* (*Lbg.*) *murinus* promoted a shift from pro-inflammatory M1 to anti-inflammatory M2 macrophages and enhanced intestinal barrier function via IL-10 release, counteracting damage induced by the environmental toxin deoxynivalenol. These findings point to bacterial EVs as key signaling vectors that can influence both gut integrity and systemic immune responses relevant to brain health.

In parallel, enteroendocrine cells (EECs) have emerged as critical sensors and transducers of microbial metabolites to the central nervous system via the vagus nerve [68]. Using a zebrafish model, it was demonstrated that tryptophan derivatives from *Edwardsiella tarda* stimulated EECs through activation of transient receptor potential ankyrin 1 (Trpa1) channels, enhancing serotonin (5-HT) release and vagal signaling. Supporting this axis, Morkl et al. [69], proposed that VN-mediated communication may underlie the beneficial effects of psychobiotics in stress related disorders. In a clinical trial involving the multi-strain probiotic OMNi-BiOTic^®^ STRESS Repair, they observed improved vagal tone, increased abundance of *Akkermansia muciniphila*, and enhanced sleep quality—further reinforcing the therapeutic potential of microbiota–vagus–brain signaling pathways.

## 4. Psychobiotics: Definition and Key Microbial Strains

Psychobiotics were initially defined as probiotic strains capable of exerting beneficial effects on brain function and behavior by acting through the GBA. As such, they share several properties with conventional probiotic strains, including non-pathogenicity (e.g., absence of hemolytic activity), catalase negativity, resistance to gastrointestinal digestion, and phenolic compounds, co-aggregation and auto-aggregation capacity, adhesion to intestinal cells, and antimicrobial activity [70].

To qualify as psychobiotics, these microorganisms must also produce neuroactive compounds, particularly neurotransmitters such as gamma-aminobutyric acid (GABA) and serotonin (5-HT), which play critical roles in mood regulation and cognitive function. These compounds can also modulate systemic inflammation and oxidative stress, both of which are closely associated with neurodegenerative diseases [71].

The selection of strains should depend on the intended biological effect. For example, in vitro studies can evaluate the potential of a strain to modulate cytokine profiles, reinforce intestinal barrier integrity, or enhance neurotransmitter synthesis. In the case of *Lactiplantibacillus plantarum* PS128, comparative genomic analysis revealed that only this strain, along with one other among several *Lpb. plantarum* strains, harbored genetic elements associated with immunomodulatory properties [72]. Such evidence highlights the value of functional genomics not just for identification, but for anticipating neuroprotective or psychotropic activity. This perspective aligns with recent frameworks proposing targeted screening pipelines for psychobiotics discovery, as discussed in previous reviews [73].

### 4.1. Preclinical Evidence

Various rodent models have been employed to investigate the effects of probiotics on the GBA. These preclinical studies consistently demonstrate that specific probiotic strains can influence behavioral outcomes, modulate neuroinflammatory responses, and alter neurotransmitter levels in the central nervous system. Both germ-free and conventionally colonized animals have proven valuable in uncovering mechanisms by which gut microbial composition impacts brain function, stress regulation, and cognitive performance [74,75,76]. Notably, colonization of germ-free mice with specific microbial consortia or individual strains has been shown to restore anxiety-like behavior, normalize HPA axis activity, and regulate expression of synaptic and inflammatory genes in the brain.

Several preclinical studies have demonstrated the ability of specific probiotic strains to modulate neurochemical and behavioral outcomes in rodent models of neuropsychiatric and neurodegenerative disorders. For instance, *Lacticaseibacillus rhamnosus* JB-1 supplementation stabilized stress-related neurometabolites such as γ-aminobutyric acid (GABA), whose imbalance is linked to anxiety and depression [77,78]. Likewise, *Lactobacillus* (*Lab.*) *helveticus* NS8 and *Lpb. plantarum* PS128 have shown to restore serotonergic, dopaminergic, and noradrenergic activity in depressive and anxiety models [77,79]. Similarly, *Bifidobacterium breve* increased hippocampal levels of indole-3-lactic acid (ILA), a tryptophan-derived metabolite associated with neuroprotection and mood regulation [77,79,80]. Moreover, the incorporation of *Lpb. plantarum* PS128 in autism spectrum disorder models has shown promising effects on behavioral improvement [74]; nevertheless, human trials are necessary to confirm its efficacy.

### 4.2. Clinical Trials

A growing number of randomized controlled trials have evaluated the efficacy of psychobiotic formulations across neurodegenerative and neuropsychiatric disorders, assessing cognitive performance, inflammatory biomarkers, and quality-of-life outcomes. Table 1 summarizes representative clinical studies, highlighting probiotic composition, study design, and principal outcomes related to Alzheimer’s disease, Parkinson’s disease, depression, and anxiety. Together, these findings provide translational evidence supporting the potential of psychobiotics as adjunctive therapies in neurological and mental health management.

### 4.3. Alzheimer’s Disease

Alzheimer’s disease (AD) is the most common cause of dementia and is characterized by progressive cognitive decline and the accumulation of β-amyloid (Aβ) and hyperphosphorylated tau aggregates [96]. Growing evidence links gut dysbiosis with neuroinflammation, oxidative stress, and blood–brain barrier disruption, all of which exacerbate neurodegeneration [97]. Altered production of SCFAs and increased trimethylamine-N-oxide (TMAO) have been implicated in protein aggregation and neuronal dysfunction, highlighting the gut microbiota’s contribution to AD pathogenesis [98,99]. These structures serve as diagnostic markers and are directly implicated in the mechanisms leading to neuronal dysfunction and death.

Clinical studies have begun to explore psychobiotic interventions as adjunct therapies for AD. Akbari et al. [81], reported improved cognitive performance and reduced serum malondialdehyde (MDA) and C-reactive protein (CRP) after 12 weeks of probiotic milk containing *Lab. acidophilus*, *Lbs. casei*, *B. bifidum*, and *L. fermentum*. Kim et al. [82], found that supplementation with *B. bifidum* BGN4 and *B. longum* BORI improved mental flexibility, reduced stress, and increased serum BDNF in elderly participants. Similarly, Hsu et al. [83], observed elevated BDNF levels following 12 weeks of treatment with a multi-strain probiotic including *B. longum* subsp. *infantis* BLI-02, *B. breve* Bv-889, *B. animalis* subsp. *lactis* CP-9, *B. bifidum* VDD088, and *Lpb. plantarum* PL-02. More recently, Jouni et al. [84], reported that *B. longum* R0175 enhanced serum amino acid profiles-precursors of key neurotransmitters, suggesting a novel metabolic pathway underlying psychobiotic effects. Collectively, these findings support the role of probiotics in modulating neurotrophic and inflammatory pathways, offering promising complementary strategies to slow cognitive decline in AD.

### 4.4. Parkinson’s Disease

Parkinson’s disease (PD) is a progressive neurodegenerative disorder marked by the selective loss of dopaminergic neurons in the substantia nigra, leading to characteristic motor symptoms such as tremor, bradykinesia, and rigidity, as well as non-motor symptoms like depression, constipation, and cognitive impairment [100,101,102,103,104].

Beyond its central pathology, emerging evidence implicates gut microbiota alterations as potential contributors to disease onset and progression [105,106,107]. Intestinal dysbiosis in PD has been associated with reduced microbial diversity and lower abundance of SCFA-producing taxa such as *Faecalibacterium* and *Lachnospiraceae* reported [108]. This imbalance may compromise intestinal barrier integrity and facilitate translocation of endotoxins like LPS, contributing to chronic systemic inflammation and α-synuclein aggregation in both the enteric and central nervous systems [109]. Fecal SCFAs levels, particularly acetate, propionate, and butyrate, are also diminished in PD, potentially affecting synaptic plasticity and neuroimmune regulation in PD [110].

Clinical trials have begun exploring probiotics as adjunct therapies for PD. In a 12-week randomized placebo-controlled study, Magistrelli et al. [85] administered a multi-strain probiotic (including *B. animalis* subsp. *lactis*, *B. longum*, and *B. adolescentis*) with fructooligosaccharides to 40 PD patients. The intervention improved both motor (UPDRS) and non-motor symptoms (NMSS), and reduced systemic IL-6 levels, suggesting anti-inflammatory effects. Other trials have demonstrated benefits in gastrointestinal symptoms, notably constipation, a common comorbidity in PD. For example, multi-strain formulations such as Hexbio^®^ (*Lbs. casei*, *Lab. delbrueckii* subsp. *lactis* (formerly *Lactobacillus lactis)*, *B. bifidum*, *B. infantis*, *B. longum*) significantly improved bowel movement frequency and stool consistency in randomized controlled trials [86,87]. Beyond symptomatic relief, probiotics may modulate immune pathways relevant to PD pathology. In a double-blind study Borzabadi et al. [88], found that 12 weeks of supplementation downregulated pro-inflammatory cytokines (IL-1, IL-8, TNF-α) and upregulated TGF-*β* and PPAR-*γ* gene expression. These findings support the potential of probiotic strategies to target multiple aspects of PD pathophysiology, from neuroinflammation to microbiota–gut–brain signaling. However, larger and longer duration clinical trials are needed to validate these benefits and optimize probiotic formulations.

### 4.5. Depression

Major depressive disorder (MDD) is a prevalent mood disorder characterized by persistent sadness, anhedonia, cognitive impairment, and feelings of hopelessness [111]. Globally, the condition affects over 23 million children and adolescents [35], with a prevalence of approximately 21% in the U.S. and 6.4% in Mexico among individuals aged 12–65 years [112]. While traditionally associated with deficiencies in serotonin (5-HT), norepinephrine (NE), and dopamine (DA), current models of depression consider a broader neurobiological interplay involving the glutamatergic and GABAergic systems, neuropeptides, and amino acid metabolism [113].

Neuroinflammation is a key mechanism implicated in depression pathophysiology. Activated microglia release pro-inflammatory cytokines, such as TNF-α, IL-1β, and IL-6, that disrupt neurotransmitter balance and neuroplasticity. Chronic stress can further exacerbate this process through activation of the hypothalamic–pituitary–adrenal (HPA) axis, resulting in glucocorticoid release and cytokine production [114,115].

The GBA is increasingly recognized as a central player in MDD. Intestinal dysbiosis can redirect tryptophan metabolism toward kynurenine production, favoring neuroinflammation and oxidative stress [116]. Increased gut permeability further permits translocation of bacterial endotoxins, triggering immune activation and interfering with 5-HT and GABA biosynthesis disorders [117]. These disruptions collectively impact mood regulation and behavior.

Clinical trials investigating psychobiotics in MDD consistently highlight their potential to modulate mood, inflammatory responses, and neuroplasticity either alone or as adjuncts to pharmacological therapy. Notably, *Lab. helveticus* R0052 and *B. longum* R0175 have been extensively studied in this context. In an early trial, Messaoudi et al. [89], administered these strains for 30 days to healthy adults and reported reduced anxiety and depressive symptoms using validated psychological scales (HADS and GSI), though no biological markers were evaluated. Building on this work, Heidarzadeh-Rad et al. [90] used the same formulation in a placebo-controlled trial involving adults already receiving antidepressant therapy. After eight weeks, participants showed not only improved depression scores (via the Beck Depression Inventory), but also increased serum levels of brain-derived neurotrophic factor (BDNF), a key molecule involved in neuroplasticity and mood regulation. These findings support the hypothesis that psychobiotics may enhance therapeutic outcomes by boosting endogenous mechanisms of neural repair. Beyond these two strains, other formulations have demonstrated beneficial immunometabolic effects. For instance, Reininghaus et al. [91], evaluated OMNi-BiOTiC^®^ Stress Repair, a multi-strain probiotic enriched with vitamin B7, in individuals with clinical depression. In addition to symptom relief, metagenomic analysis of stool samples revealed upregulation of inflammation-regulatory pathways, particularly those involving IL-17, a cytokine implicated in both gut and brain inflammation. This study underscores the microbiome’s potential as both a therapeutic target and biomarker source. Similarly, Zhu et al. [92] explored the effects of *Lpb plantarum* JYLP-326 in a cohort of university students experiencing anxiety and depressive symptoms. Over three weeks, participants who received the probiotic reported improvements in mood, anxiety, and sleep quality. Microbiota profiling revealed reduced abundance of *Bacteroides*, *Faecalibacterium*, and *Roseburia*, genera often associated with low-grade systemic inflammation. Recent multi-omics studies suggest that microbiota-mediated proline metabolism may play a causal role in depression through its impact on glutamate and GABA signaling in the brain. Elevated circulating proline, linked to extracellular matrix remodeling and altered gene expression in the prefrontal cortex, has been associated with depressive symptoms in both humans and microbiota-transplanted mice. These findings support the idea that dietary modulation of amino acids like proline, alongside targeted psychobiotics (e.g., *Lpb. plantarum*, a GABA producer), could influence mood-related neurotransmission via the gut–brain axis and warrant further exploration in preclinical models [118].This reinforces the concept that gut microbiota modulation can influence behavioral outcomes in at-risk but otherwise healthy populations.

Together, these studies suggest a convergent psychobiotics effect across different populations and formulations: improved mood symptoms, regulation of inflammatory markers, modulation of neurotrophic factors, and reshaping of gut microbial composition. However, further trials with standardized endpoints and larger sample sizes are needed to clarify strain-specific mechanisms and optimize clinical application.

### 4.6. Anxiety and the GBA

Anxiety is a multifactorial and often debilitating mental health disorder, characterized by persistent worry, anticipatory fear, and, in some cases, panic attacks. Globally, it ranks among the most prevalent psychiatric conditions, affecting an estimated 58 million children and adolescents worldwide, with lifetime prevalence rates of 31% in the United States and 7.9% in Mexico [112,119,120].

At the neurobiological level, anxiety involves dysregulation of key neurotransmitter systems, particularly gamma-aminobutyric acid (GABA), serotonin (5-HT), norepinephrine (NE), and glutamate, along with their corresponding receptors. The amygdala plays a central role in fear processing, and its hyperactivity has been implicated in the exaggerated stress responses characteristic of anxiety disorders [121]. Additionally, these disorders have been linked to inflammation and anxiety through signaling molecules (cytokines) released by microglia and astrocytes to limbic structures and amygdalae, provoking exaggerated fear responses [122].

Clinical evidence supports the potential role of probiotics in alleviating anxiety symptoms, particularly through immune-neuroendocrine modulation. For instance, Tran et al. [93], investigated various combinations of Lactobacilli and *Bifidobacterium* species in a population of college students. Following a 28-day intervention, participants reported reduced anxiety scores, suggesting a general anxiolytic effect of multi-strain formulations.: *B. lactis*, *B. bifidum*, *B. breve*, *B. longum*, *B. infantis*, *B. adolescentis*, *Lab. acidophilus*, *Levilactobacillus* (*Lvb.*) *brevis*, *Lab. delbrueckii* subsp. *bulgaricus* (formerly *Lactobacillus bulgaricus*), *Lbs. casei*, *Lbs. paracasei*, *Lpb. plantarum*, *Lgb. salivarius*, *Lbs. rhamnosus*, *Lmb. fermentum*, *Lab. crispatus*, *Lab. gasseri*, *Lab. helveticus*, *Lmb. reuteri*, *Lactococcus lactis*, *Streptococcus thermophilus* or *Bacillus coagulans*

Expanding on these findings, Walden et al. [94], conducted a randomized, double-blind, placebo-controlled trial using a defined multi-strain mixture (*Lpb. plantarum* LP01, *Lmb. fermentum* LF16, *Lbs. rhamnosus* LR06, and *B. longum* 04). Participants not only showed improved mood and reduced anxiety according to validated scales, but also exhibited significantly higher plasma serotonin levels and reduced cortisol concentrations, highlighting a possible neuroendocrine mechanism of action.

Further supporting this, Ma et al. [95], demonstrated that 12-week supplementation with *Lpb. plantarum* P-18 not only improved anxiety and depressive symptoms but also modulated the gut metagenome at the level of species-genome bins (SGBs) and functional gene expression. These findings suggest that probiotic efficacy may depend not only on microbial diversity but also on specific microbial functions.

## 5. Future Perspectives in Psychobiotics Therapies

The therapeutic use of probiotics and psychobiotics has shown promising effects across various neurodegenerative and neuropsychiatric conditions, such as Alzheimer’s disease (AD), Parkinson’s disease (PD), major depressive disorder (MDD), and anxiety. Beyond conventional probiotic approaches, recent advancements are shaping the future of this field. One innovation is the use of synbiotics (combinations of probiotics with prebiotics) that synergistically enhance bacterial survival and proliferation. These formulations can modulate the microbiota more robustly and have been linked to improved metabolic and neurochemical markers in various models. Next-generation probiotics represent a major frontier. Unlike traditional strains derived from fermented foods (e.g., lactobacilli, bifidobacteria), next-generation candidates like *Akkermansia muciniphila* (first isolated in 2004). Nowadays, many animal trials have been performed and results encourage its use to treat obesity, cancer, and depression [123,124,125]. Precision psychobiotics, another emerging approach, combine microbiome profiling, targeted strain selection, and genome sequencing with biomarker-driven clinical designs. These tools aim to optimize therapeutic outcomes by matching psychobiotic interventions with individual host profiles.

The potential for psychobiotics to address dual pathologies is especially relevant given the frequent comorbidity of neurological and psychiatric conditions. For instance, in PD, patients may also experience early cognitive decline or depression, while gut microbiota alterations can exacerbate these non-motor symptoms [85,126]. Psychobiotics could offer a route to modulate overlapping mechanisms (like GABA and serotonin signaling) across disorders. Importantly, microbial interventions might also enhance standard pharmacological treatments. In PD, for example, gut microbes can alter levodopa availability, but microbial modulation (e.g., *H. pylori* eradication) has improved drug response [126]. Thus, co-therapy strategies integrating probiotics with conventional treatments may enhance therapeutic efficacy in complex cases.

As evidence grows, integrating psychobiotics into clinical care will require clarity on strain specificity, host factors, and disease stage. Future directions include personalized psychobiotic formulas, co-therapy models, and broader inclusion in multimodal treatment plans.

### 5.1. Challenges in Clinical Translation

Despite growing evidence supporting the role of psychobiotics in modulating the gut–brain axis, their clinical translation faces several unresolved challenges. Neuroinflammation and cognitive dysfunction continue to represent major barriers in neurological and psychiatric care, diminishing quality of life and complicating long-term management. Findings from both animal and clinical studies underscore that systemic and localized inflammation, often triggered by infections or chronic stressors, can impair memory, learning, and mood regulation [127,128]. In epilepsy, for instance, over 80% of patients experience depressive symptoms, revealing the tight interplay between neuroinflammation and affective comorbidities [129]. These observations further align with mechanistic insights that implicate inflammation in the pathophysiology of depression and its social and functional consequences [130].

### 5.2. Strain Selection, Standardization, and Biomarkers

A central challenge in the clinical deployment of psychobiotics lies in the precise selection of microbial strains. The efficacy of probiotics is highly strain-dependent, with even closely related strains exhibiting divergent effects. For example, *Lbs. casei* DN-114001 may reduce antibiotic-associated diarrhea, whereas *Lbs. rhamnosus* GG may not; similarly, *Saccharomyces boulardii* CNCM I-745 is effective in irritable bowel syndrome, but not all *S. cerevisiae* strains are interchangeable [131].These differences stem from specific microbial properties, such as modulation of host immunity, metabolite production, and interaction with the existing microbiota.

Standardization remains equally critical. Host factors, including baseline microbiota composition, dietary patterns, and immune status, introduce variability that complicates reproducibility across trials. Moreover, evaluating clinical efficacy demands robust, validated biomarkers. Current efforts focus on quantifying neuroactive metabolites (e.g., GABA, serotonin, dopamine), pro-inflammatory cytokines (e.g., IL-6, TNF-α), and gut integrity markers (e.g., zonulin), often via ELISA, mass spectrometry, or liquid chromatography [132].Yet, detection sensitivity and the invasive nature of sampling limit their widespread use. Future strategies should prioritize minimally invasive techniques, coupled with well-powered trials using rigorously selected strains.

### 5.3. Lifestyle and Dietary Modulators

The clinical effectiveness of psychobiotics cannot be fully understood without considering lifestyle and diet. Gut microbiota is profoundly shaped by dietary habits, which in turn influence psychobiotics colonization and activity. Berding et al. [133] demonstrated that a diet rich in prebiotics and fermented foods can enhance microbial stability and positively influence perceived stress levels in healthy adults. Similarly, plant-based diets high in bioactive compounds have been linked to improved mental health outcomes, reinforcing the idea that dietary diversity potentiates psychobiotic effects [133,134]. Timing also matters. For example, the administration of *Lmb. fermentum* PS150™ has shown sleep-promoting effects in mice when synchronized with the circadian rhythm [134], suggesting that psychobiotic efficacy may depend on both the strain and its temporal context. Moreover, regular physical activity and stress management (e.g., mindfulness, meditation) have been associated with positive shifts in gut microbial composition and may synergize with psychobiotics interventions Morales-Torres et al. [135]. Beyond symptomatic relief, these lifestyle factors may enhance the preventive potential of psychobiotics in neurodegenerative contexts, where inflammation and oxidative stress play central roles [136].

### 5.4. Ethical and Methodological Considerations

Ethical integrity and methodological rigor are non-negotiable in psychobiotic trials. Clear and comprehensible informed consent is essential, especially when working with cognitively vulnerable populations [137]. Study designs must be robust enough to isolate treatment effects while minimizing bias. While randomized controlled trials (RCTs) remain the gold standard, heterogeneity in psychobiotic dosing, duration, and lifestyle control continues to limit comparability.

Furthermore, integrating lifestyle variables into research introduces complexity. Aththanayaka [138] emphasized that psychobiotics effects cannot be disentangled from behavioral contexts such as diet and exercise. Therefore, future clinical frameworks must adopt a more holistic approach that includes microbiome profiling, behavioral tracking, and standardized outcome measures emphasizes the importance of clear communication, particularly when involving vulnerable populations, such as older adults, who may have cognitive impairments that affect their understanding of the consent process.

Methodological rigor is crucial for the validity of clinical studies. The design of studies involving psychobiotics must be carefully considered to minimize bias and ensure reliable outcomes. For instance, randomized controlled trials (RCTs) are often regarded as the gold standard for evaluating the efficacy of interventions. Morales-Torres et al. [135] discuss how lifestyle behaviors can modulate the effects of psychobiotics, which highlights the need for consistent study protocols to enhance robustness.

## 6. Conclusions

Psychobiotics have emerged as a promising adjunctive strategy in the management of neuropsychiatric and neurodegenerative disorders. Their ability to influence mood, cognition, and systemic inflammation through gut–brain communication is supported by growing experimental and clinical evidence. Notably, neuroinflammation and oxidative stress remain central drivers of cognitive decline and emotional dysregulation. In this context, psychobiotics, when paired with lifestyle modifications such as a diverse diet, physical activity, and stress reduction, may contribute to restoring homeostasis within the gut–brain axis.

The integration of gut microbiota–related biomarkers into clinical protocols offers an avenue to improve patient stratification and monitor treatment response with greater precision. However, translational challenges persist, particularly in terms of strain specificity, standardization, and the influence of host variability. Addressing these hurdles will require methodologically sound clinical trials that incorporate robust endpoints, including neuroactive metabolites, inflammatory markers, and cognitive assessments.

Ethical rigor and interdisciplinary collaboration are equally important for advancing psychobiotics toward evidence-based application. As the field moves forward, combining microbiota-targeted strategies with conventional pharmacotherapy and precision medicine frameworks may unlock novel neuroprotective interventions and contribute to improved mental health outcomes across the lifespan.

## Figures and Tables

**Table 1:** Summary of Clinical Trials Evaluating the Effects of Psychobiotics on Neuropsychiatric and Neurodegenerative Disorders.

Condition	Probiotic Strain(s)	Study Design	Reported Outcomes	Reference
Alzheimer’s Disease	*Lab. acidophilus*, *Lbs. casei*, *B. bifidum*, *Lmb. Fermentum*	Double-blind, RCT, 12 weeks	↑ Cognition (MMSE), ↓ hs-CRP and MDA	[81]
Alzheimer’s Disease	*B. bifidum* BGN4, *B. longum* BORI	RCT, 63 elderly adults	↑ Mental flexibility, ↑ serum BDNF, ↓ pro-inflammatory taxa	[82]
Alzheimer’s Disease	*B. longum* subsp. *infantis* BLI-02, *B. breve* Bv-889, *B. animalis* subsp. *lactis* CP-9, *B. bifidum* VDD088, *Lpb. plantarum* PL-02	Double-blind, RCT, 12 weeks	↑ BDNF levels, improved cognitive measures	[83]
Alzheimer’s Disease	*Lbs. rhamnosus* HA-114, *B. longum* R0175	RCT, 12 weeks	↑ Serum amino acid profile; neurotransmitter precursor relevance	[84]
Parkinson’s Disease	*B. lactis* BS01, *B. longum* BL03, *B. adolescentis* BA02 + FOS	RCT, 12 weeks	↑ Motor and non-motor scores, ↓ IL-6, ↑ TGF-β	[85]
Parkinson’s Disease	*Lab. acidophilus*, *Lbs. casei*, *Lab. Delbrueckii* subsp. *lactis*, *B. bifidum*, *B. infantis*, *B. longum* (Hexbio^®^, B-Crobes Laboratory Sdn Bhd, Ipoh, Malaysia)	2 RCTs	↑ Bowel movements, ↓ gut transit time	[86,87]
Parkinson’s Disease		RCT, 12 weeks	↓ IL-1, IL-8, TNF-α; ↑ TGF-β, PPAR-γ	[88]
Depression	*Lab. helveticus* R0052, *B. longum* R0175	Double-blind, placebo-controlled, 30 days	↓ Anxiety and depression (HADS, GSI)	[89]
Depression	*Lab*. *helveticus* R0052, *B*. *longum* R0175	RCT, 8 weeks + pharmacotherapy	↓ BDI scores, ↑ BDNF	[90]
Depression	OMNi-BiOTiC^®^ Stress Repair (a multi-strain probiotic, Institute AllergoSan, Graz, Austria)	Double-blind RCT, 28 days	↓ Symptoms, ↑ IL-17 pathway modulation	[91]
Depression	*Lpb. plantarum* JYLP-326	RCT, 3 weeks in anxious students	↓ Depression, anxiety, insomnia; ↓ *Bacteroides, Faecalibacterium*, *Roseburia*	[92]
Anxiety	Multi-strain: *B. lactis*, *Lab. acidophilus*, *Lbs. rhamnosus*, etc.	RCT, 28 days (students)	↓ Anxiety (questionnaires)	[93]
Anxiety	*Lpb. plantarum* LP01, *Lmb. fermentum* LF16, *Lbs. rhamnosus* LR06, *B. longum* 04	RCT, 28 days	↑ Serotonin, ↓ CRP, ↑ mood	[94]
Anxiety	*Lpb. plantarum* P-18	RCT, 12 weeks	↓ Anxiety and depression, microbiome SGB enrichment	[95]

Abbreviations: BDNF = Brain-derived neurotrophic factor; MMSE = Mini-Mental State Examination; MDA = Malondialdehyde; hs-CRP = High-sensitivity C-reactive protein; HADS = Hospital Anxiety and Depression Scale; GSI = Global Severity Index; BDI = Beck Depression Inventory; IL-6 = Interleukin 6; TGF-β = Transforming growth factor beta; CRP = C-reactive protein; SGB = species-genome bins; Arrows indicate increases (↑) or decreases (↓) relative to the control group.

## Data Availability

No new data were created or analyzed in this study. Data sharing is not applicable to this article.
